# Higher Balance Task Demands are Associated with an Increase in Individual Alpha Peak Frequency

**DOI:** 10.3389/fnhum.2015.00695

**Published:** 2016-01-06

**Authors:** Thorben Hülsdünker, Andreas Mierau, Heiko K. Strüder

**Affiliations:** Institute of Movement and Neurosciences, German Sport University CologneCologne, Germany

**Keywords:** EEG, stance, posture, theta, iAPF, sensorimotor, cortex, error detection

## Abstract

Balance control is fundamental for most daily motor activities, and its impairment is associated with an increased risk of falling. Growing evidence suggests the human cortex is essentially contributing to the control of standing balance. However, the exact mechanisms remain unclear and need further investigation. In a previous study, we introduced a new protocol to identify electrocortical activity associated with performance of different continuous balance tasks with the eyes opened. The aim of this study was to extend our previous results by investigating the individual alpha peak frequency (iAPF), a neurophysiological marker of thalamo-cortical information transmission, which remained unconsidered so far in balance research. Thirty-seven subjects completed nine balance tasks varying in surface stability and base of support. Electroencephalography (EEG) was recorded from 32 scalp locations throughout balancing with the eyes closed to ensure reliable identification of the iAPF. Balance performance was quantified as the sum of anterior-posterior and medio-lateral movements of the supporting platform. The iAPF, as well as power in the theta, lower alpha and upper alpha frequency bands were determined for each balance task after applying an ICA-based artifact rejection procedure. Higher demands on balance control were associated with a global increase in iAPF and a decrease in lower alpha power. These results may indicate increased thalamo-cortical information transfer and general cortical activation, respectively. In addition, a significant increase in upper alpha activity was observed in the fronto-central region whereas it decreased in the centro-parietal region. Furthermore, midline theta increased with higher task demands probably indicating activation of error detection/processing mechanisms. IAPF as well as theta and alpha power were correlated with platform movements. The results provide new insights into spectral and spatial characteristics of cortical oscillations subserving balance control. This information may be particularly useful in a clinical context as it could be used to reveal cortical contributions to balance dysfunction in specific populations such as Parkinson’s or vestibular loss. However, this should be addressed in future studies.

## Introduction

The maintenance of balance is essential for the majority of motor activities in daily life. This includes rather automated processes such as the maintenance of upright posture as well as more complex movements during sports or when balance is disrupted unpredictably. For long, balance has been assumed to be controlled by subcortical structures of the cerebrum, the spinal cord, and the cerebellum (Keck et al., 1998). However, there is growing evidence indicating the involvement of cortical structures during the control of balance and posture (Ouchi et al., 1999; Slobounov et al., 2005, 2009; Jacobs and Horak, 2007; Maki and McIlroy, 2007; Mihara et al., 2008; Bolton et al., 2012; Mierau et al., 2015b). Previous studies using electroencephalography (EEG) during balance perturbations revealed higher demands on balance control are associated with higher theta activity located in the medial fronto-central cortical area including the anterior cingulate cortex (ACC) (Slobounov et al., 2009, 2013; Varghese et al., 2014). As the ACC has previously been shown to be involved in error-detection and processing during cognitive tasks (Luu et al., 2004; Trujillo and Allen, 2007), it is suggested to subserve a similar process during balance control by detecting discrepancies between expected and actual state of balance (Adkin et al., 2006; Slobounov et al., 2009; Mochizuki et al., 2010). Balance-related increases in theta activity are, however, not restricted to the fronto-central cortex but also include centro-parietal regions (Sipp et al., 2013; Hülsdünker et al., 2015) which are well-known to be crucially involved in multimodal sensory integration and sensorimotor coordination (Andersen et al., 1997).

The cumulative pattern of results obtained in the aforementioned studies addressing cortical activity during balance control suggests increased allocation of cortical resources in more demanding balance situations. However, it remains unclear if this is attributable to an overall increase of information that reaches the cortex when balance tasks become more challenging. To address this question, additional analysis of frequency-specific oscillatory components such as the individual Alpha Peak Frequency (iAPF) is recommended. The iAPF has been defined as the maximal power value in the frequency spectrum between 8 and 12 Hz (Klimesch, 1999), and is best detectable during eyes-closed conditions. The alpha rhythm consists of inhibitory and excitatory phases that are proposed to frame information transmission between the cortex and the thalamus (Lorincz et al., 2009). Accordingly, higher iAPF would increase the number of excitation-inhibition cycles per time and thus, support information flow to the cortex. Therefore, while task-related modulations in spectral power, the main measure in previous studies, are suggested to reflect adjustments in cortical resource investment, alterations in iAPF reflect an upstream process regulating the overall amount of information that reaches the cortex. Consistent with this, recent studies revealed significant increases in iAPF during performance of more challenging cognitive tasks indicating an increase of information transmission when a task becomes more demanding (Haegens et al., 2014; Maurer et al., 2014). We argue this mechanism is not limited to purely cognitive tasks but should also apply to the sensorimotor domain.

The main purpose of the present study was to extend previous research addressing balance-related modulations in spectral power by investigating the iAPF in the context of balance control. To this end eyes-closed balance conditions were used to ensure valid and reliable assessment of iAPF (Bazanova, 2011). The iAPF is hypothesized to increase with higher demands on balance control, and this is suggested to reflect an increase of the amount of information relayed to the cortex via the thalamus (Lorincz et al., 2009). Testing this hypothesis is important because spectral power and iAPF reflect different processes in the brain (see above). Therefore, analyzing the iAPF carries a great potential to gain new insights into the cortical mechanisms underlying balance control and probably other sensorimotor tasks.

Another purpose of the present study is to extend the current literature dealing with cortical activity associated with continuous balance task performance. Specifically, in a previous study we reported theta power modulations as a function of balance task demands during eyes-open conditions (Hülsdünker et al., 2015). Here, we aimed to do identical analyses, however, during eyes-closed balance conditions to better understand the role of visual information processing on balance-related spectral power modulations. In addition to theta power, activity in the lower (8–10 Hz) and the upper (10–12 Hz) alpha frequency bands was also analyzed as the alpha band has also been shown to play an important role in balance control (Del Percio et al., 2009; Slobounov et al., 2009). We expected an increase of theta power in fronto-central and centro-parietal regions with increasing balance task demands, and this is suggested to reflect an increased magnitude/amount of errors that have to be detected and processed during performance of more demanding tasks. In contrast to theta power, alpha power should be reduced indicating greater cortical activation.

## Materials and Methods

### Subjects and Ethics

Thirty-nine healthy male university students participated in the study. The study was subdivided into two independent experiments investigating balance tasks under eyes-opened and eyes-closed conditions, respectively. The order of eyes-opened and eyes-closed conditions was counterbalanced across subjects. Subsequently, a third perturbation experiment was conducted. The results on eyes-opened balance tasks and the perturbation experiment have been reported previously (Hülsdünker et al., 2015; Mierau et al., 2015b). The results on eyes-opened balance tasks and the perturbation experiment have been reported previously (Hülsdünker et al., 2015; Mierau et al., 2015b). In two subjects, the applied artifact detection algorithm prior to independent component analysis (ICA) detected artifacts in more than 80% of all segments within several balance conditions; therefore, these subjects were excluded from further analyses. The remaining 37 participants (age: 24.7 ± 3 years; body weight: 77.3 ± 8.1 kg; height: 180.4 ± 5.1 cm; body mass index: 23.8 ± 2.4) confirmed being free of injury for at least 6 months, having no pain or discomfort and/or experiencing any limitation in the range of motion during their daily routine and physical activity. In addition, all participants confirmed they did not undertake physical exercise in the 48 h prior to the test. They were informed about the experimental protocol and their written consent was obtained. The study was reviewed and approved by the local ethics committee of the university in accordance with the declaration of Helsinki.

### Experimental Protocol

Prior to the experiment, the subject’s dominant leg was identified by applying the inventory of Reiss and Reiss (2000). The experimental protocol was similar to our previous study (Hülsdünker et al., 2015) comprising nine balance tasks with a systematic modulation of balance control demands (see **Table 1**). Each participant performed the balance tasks while standing in an upright position on the Posturomed^®^ (Haider Bioswing, Pullenreuth, Germany).

**Table 1:** 3 × 3 factorial design of the nine balance tasks.

Base of support (BOS)	Surface stability (SUS)		
	
	Stable surface	Instability level 1	Instability level 2
Bipedal	BOS1 – SUS1	BOS1 – SUS2	BOS1 – SUS3
Unipedal: dominant leg	BOS2 – SUS1	BOS2 – SUS2	BOS2 – SUS3
Unipedal: non-dominant leg	BOS3 – SUS1	BOS3 – SUS2	BOS3 – SUS3


*BOS1, bipedal stance; BOS2, unipedal dominant leg; BOS3, unipedal non-dominant leg; SUS1, stable surface; SUS2, instability level 1; SUS3, instability level 2.*

**FIGURE 1:**
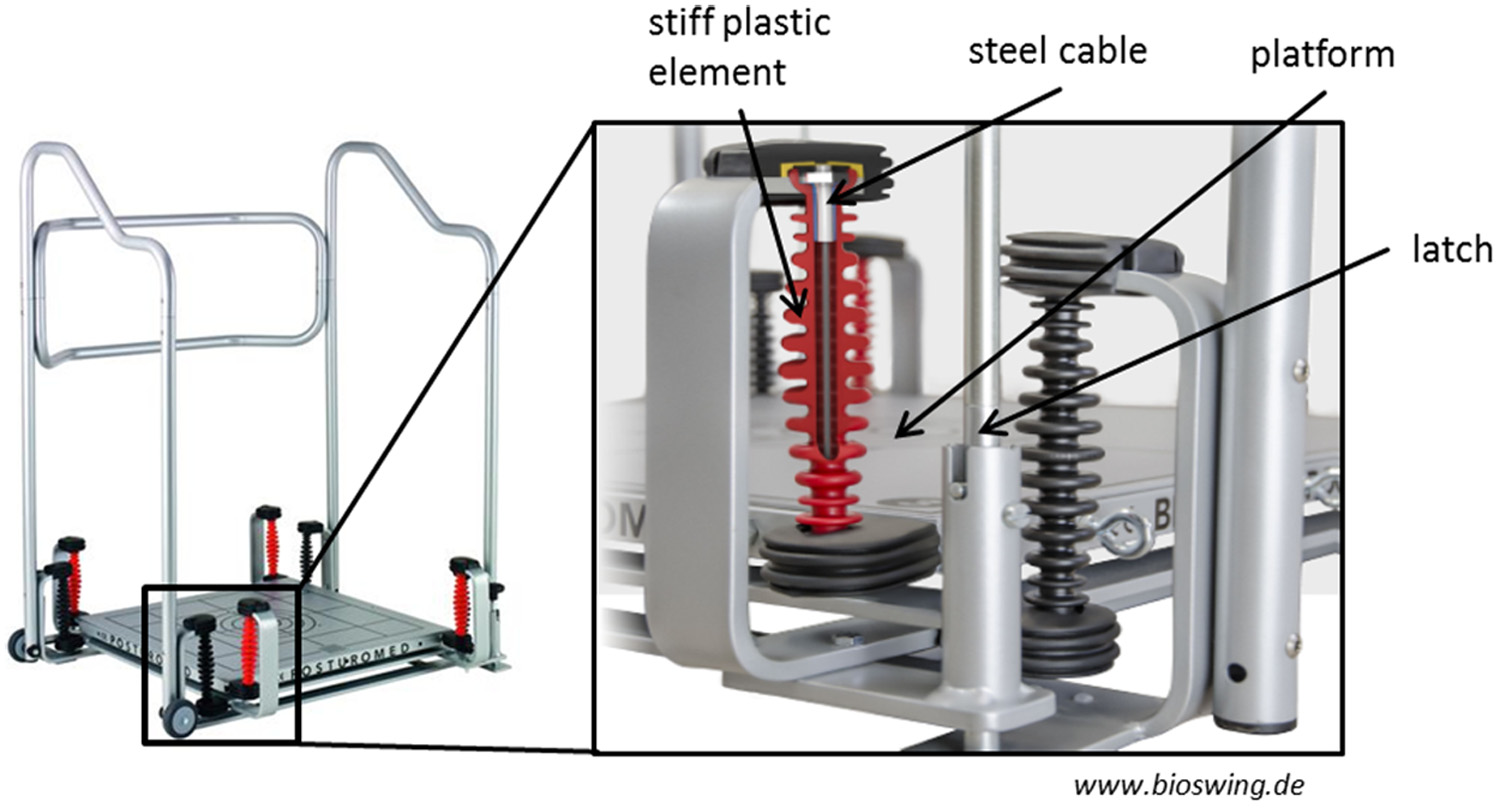
**The Posturomed.** The mechanical properties that regulate the amount of possible platform movements (steel cables, stiff plastic elements, latch system) are enlarged. With permission from HAIDER BIOSWING^®^.

The Posturomed (**Figure 1**) is a platform (60 cm × 60 cm) which is freely suspended on a frame with two oscillators, and it is characterized by a progressively attenuated oscillation behavior in response to external forces (i.e., muscular force). This means that the attenuation and thus, the deflection resistance is exponentially growing with increasing deflection of the platform due to stiff plastic elements that envelop the steel cables used to mount the oscillation frame. The mechanical constraints, validity and reliability of the Posturomed system have been described in several previous studies (Müller et al., 2004; Boeer et al., 2010a,b).

The nine tasks differed in difficulty by altering the factors “surface stability (SUS)” (SUS1, stable surface; SUS2, instability level 1; SUS3, instability level 2) and “base of support (BOS)” (BOS1, bipedal; BOS2, unipedal dominant leg; BOS3, unipedal non-dominant leg). The subjects BOS varied according to stance position (bipedal or unipedal). SUS was altered as follows. The movement amplitude and the movement frequency at which the Posturomed is deflected depend on the locking or the release of the oscillators. The largest movement amplitude occurs when the latch (see **Figure 1**) is open and thus, both oscillators are released (i.e., instability level 2). The movement amplitude is reduced if the latch is closed; i.e., only one oscillator is released (i.e., instability level 1). The higher the movement amplitude of the platform, the higher the possible amplitude of the center of pressure, and the more demanding the stabilization of the body in the respective support surface. In the “stable surface” condition all tasks were performed on the floor of the laboratory.

Balance tasks were counterbalanced across the subjects. Subjects were instructed to place their foot/feet on predefined markers in order to ensure reproducibility. For each balance task 60 s of data was recorded. To reduce artifacts subjects were instructed to place their hands at the hips and to avoid head movements. To ensure a consistent head position across balance tasks, subjects view a fixation cross at eye level after entering the platform. Three seconds prior to each trial, subjects were then instructed to close their eyes while keeping head position. During SUS1, subjects stood on the floor of the laboratory. During SUS2 and SUS3 the SUS was altered using the latch.

### Data Acquisition

#### Platform Movements

Balance performance was assessed by means of platform movements of the Posturomed. These were recorded in anterior-posterior (*y*) and medial–lateral (*x*) direction using a non-contact inductive measurement system mounted underside. This system was calibrated before each trial. The corresponding software provides the *x* and *y*-coordinates of the platform with 100 Hz temporal and 0.1 mm spatial resolution.

#### Electroencephalography

Electroencephalography was recorded from 32 scalp locations (Brain Products GmbH, Munich, Germany) overlying the whole scalp (Fp1, Fp2, F7, F3, Fz, F4, F8, FC5, FC1, FCz, FC2, FC6, T7, C3, C1, Cz, C2, C4, T8, CP5, CP1, CPz, CP2, CP6, P7, P3, Pz, P4, P8, O1, Oz, O2) and equally distributed over both hemispheres according to the international 10:10-system (Jurcak et al., 2007). One additional electrode was used to measure electrooculographic signals. The electrical reference and the ground electrode were located on position FCz and AFz, respectively. The sampling rate was set to 1000 Hz. Electrode impedances were kept below 5 kΩ.

### Data Analysis

#### Platform Movements

The total magnitude of platform movements for each balance task was quantified as the sum of absolute (modulus) anterior-posterior and medial–lateral deflections.

#### Electroencephalography

##### Pre-processing

Electroencephalography data were analyzed using the Brain Vision Analyzer 2 software (Brain Products, Munich, Germany). Raw data were first band-pass filtered (2–120 Hz) and segmented into epochs of one second. A semiautomatic algorithm was used to exclude channels and/or segments contaminated by artifacts. For the remaining segmented data of each balance condition an extended runica algorithm implemented in EEGLAB (Delorme and Makeig, 2004) was used for ICA decomposition. Based on cortical mapping, frequency spectrum and time course, components representing ocular or muscular artifacts were identified by visual inspection and removed from the data. The average number of removed components per subject was 8 ± 0.8. After ICA back transform, data were re-referenced to a common average reference.

##### Spectral power

To investigate balance related changes in cortical activity, power values for the theta (4–7 Hz), lower alpha (8–10 Hz) and upper alpha (11–13 Hz) frequency bands were calculated using fast Fourier transformation (FFT; frequency resolution: 1 Hz, 10% Hanning window).

##### Individual alpha peak frequency

**FIGURE 2:**
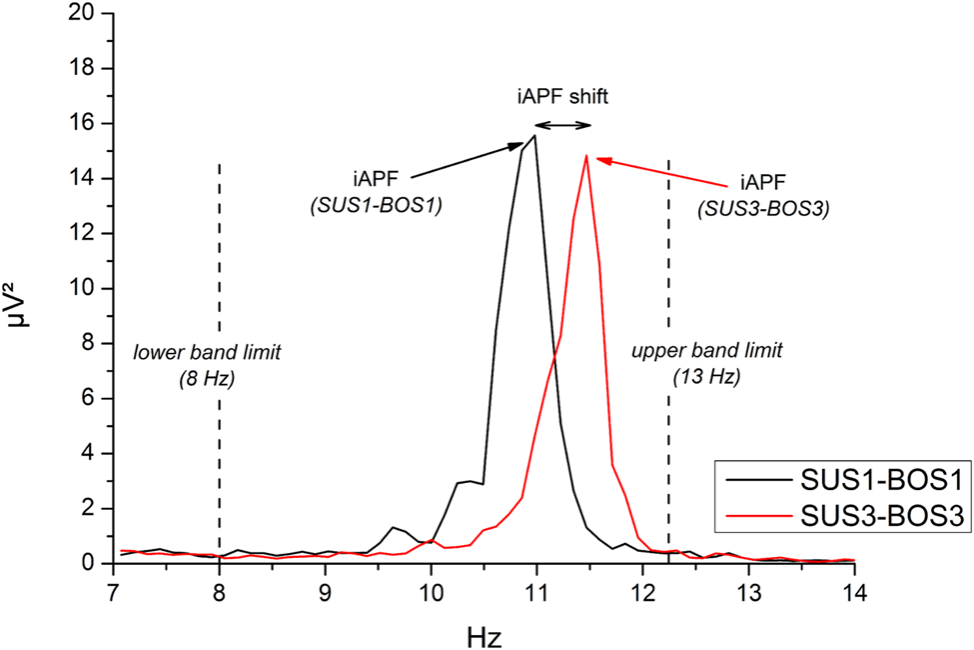
**Example of individual alpha peak frequency (iAPF) at electrode site Oz during SUS1-BOS1 (black line) and SUS3-BOS3 (red line) of one representative subject.** iAPF is defined as the maximal power value across frequency bins in the frequency range from 8 Hz (lower band limit) to 13 Hz (upper band limit).

To determine modulations in iAPF across balance tasks, data were segmented into epochs of 5 s (frequency resolution: 0.2 Hz, 10% Hanning window). The iAPF was determined for each balance task and each electrode position based on the frequency spectrum of the FFT. Specifically, the iAPF has been defined as the frequency bin (frequency resolution = 0.2 Hz) indicating the largest power value within the frequency range 8–13 Hz. The iAPF was considered as “peak frequency” only if its power exceeded the value at the preceding and the subsequent frequency bin. An example of iAPF and a balance task-related shift are shown in **Figure 2**.

We did not choose the alpha bands based on iAPF because the length of the segments and thus, the frequency resolution, was different for the iAPF and spectral power analyses. Specifically, for iAPF determination we used 5 s epochs in order to achieve a frequency resolution of 0.2 Hz. This is necessary as the individuals’ change in iAPF in response to stimuli may lie well below 1 Hz (Haegens et al., 2014; Gutmann et al., 2015). However, such high frequency resolution (i.e., long segments) comes at the cost of the number of segments available for the average procedure. Because of this inevitable tradeoff, the length of the epochs should ideally be adjusted to the purpose of the experiment. In the present study a frequency resolution of 1 Hz was sufficient for spectral power analyses and therefore, 1 s epochs were chosen in order to generate a larger number of segments that form the grand average FFT.

##### Regions of interest

According to previous studies (Slobounov et al., 2008; Hülsdünker et al., 2015), regions of interest (ROI) were defined as follows: frontal (F3, Fz, F4), fronto-central (FC1, FCz, FC2, C3, C1, Cz, C2, C4) and centro-parietal (CP1, CPz, CP2, P3, Pz, P4). These ROIs are suggested to represent cortical areas associated with cognitive, pre-motor/motor, and somatosensory processing, respectively (Koessler et al., 2009). Prior to statistical analysis the natural logarithm was applied to the absolute power values to approximate normal distribution (Gasser et al., 1982).

### Statistical Analysis

All statistical comparisons were conducted in Statistica 7.1 (StatSoft, Tusla, OK, USA). Platform movements were analyzed using a two-way repeated measurement analysis of variance (ANOVA) with the within subject factors SUS (SUS2, SUS3) and BOS (BOS1, BOS2, BOS3). EEG spectral power and iAPF were analyzed using a three-way ANOVA with the within-subject factors SUS (SUS1, SUS2, SUS3), BOS (BOS1, BOS2, BOS3), and ROI (frontal, fronto-central, centro-parietal) for each frequency band, respectively. The sphericity assumption was evaluated using the Mauchly’s test and Greenhouse–Geisser correction of degrees of freedom was used in case of non-sphericity. Bonferroni correction was used to account for multiple *post hoc* comparisons. Effect sizes were calculated using partial eta square (η^2^).

To examine the relationship between platform movements and theta power, we first calculated a general linear model ANOVA with cortical activity as a dependent variable, platform movements as a predictor variable and subject as a categorical factor. In a next step, correlation coefficients were determined based on the ANOVA results (Bland and Altman, 1995). Variability between subjects as defined by the categorical factor “subject” was removed from the analysis. According to equation 1 (Eq. 1) correlation coefficients (*r*) were then calculated based on the sum of squares (SS) for platform movements and the residuals.

$$r=\sqrt{\frac{SS\left(platform\,\;movements\right)}{SS(platform\,\;movements)+SS\left(residual\right)}}$$

As electrodes located along the fronto-central and centro-parietal cortical midline exhibited the most pronounced changes in cortical activity as a function of balance task demands, the focus of the correlation analyses was set on electrode positions FCz, Cz, CPz, Pz.

## Results

The results section presents statistical outcome variables for all comparisons. The underlying means and 95% confidence intervals of oscillatory components (ln-transformed frequency band power, iAPF) and platform movements for all balance tasks are presented in **Supplementary Table S1**.

### Platform Movements

**FIGURE 3:**
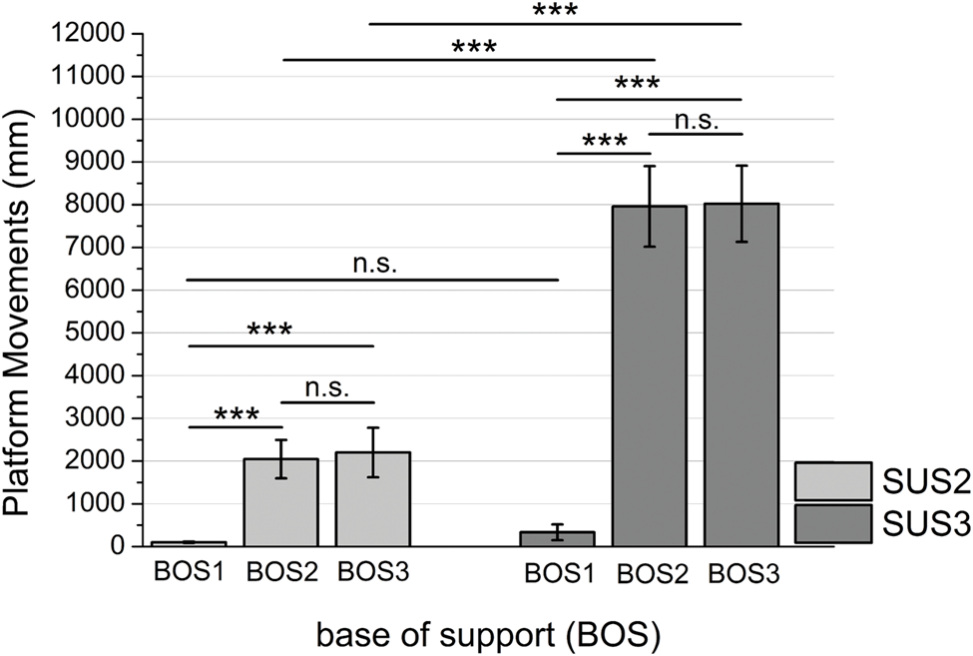
**Mean platform movements indicated for BOS1 (bipedal stance), BOS2 (unipedal stance, dominant leg), and BOS3 (unipedal stance, non-dominant leg) during SUS2 (instability level 1) and SUS3 (instability level 2).** Significance levels for *post hoc* tests are defined as follows: not significant (n.s.), ^∗∗∗^*p* < 0.001. Error bars indicate 95% confidence intervals.

Mean platform movements are presented in **Figure 3**. The ANOVA revealed significant main effects for SUS (*F*_1,36_ = 307.49, *p* < 0.001; η^2^ = 0.89) and BOS (*F*_2,72_ = 204.32, *p* < 0.001; η^2^ = 0.84) as well as a significant SUS × BOS interaction (*F*_2,72_ = 259.50, *p* < 0.001; η^2^ = 0.88). *Post hoc* analyses revealed platform movements increased during instability level 2 (SUS3) when compared to instability level 1 (SUS2) as well as during unipedal stance (BOS2, BOS3) when compared to bipedal stance (BOS1). *Post hoc* analysis of the SUS × BOS interaction indicated a stronger increase in platform movements from bipedal stance (BOS1) to unipedal stance (BOS2, BOS3) during SUS3 when compared to SUS2.

### Individual Alpha Peak Frequency

**FIGURE 4:**
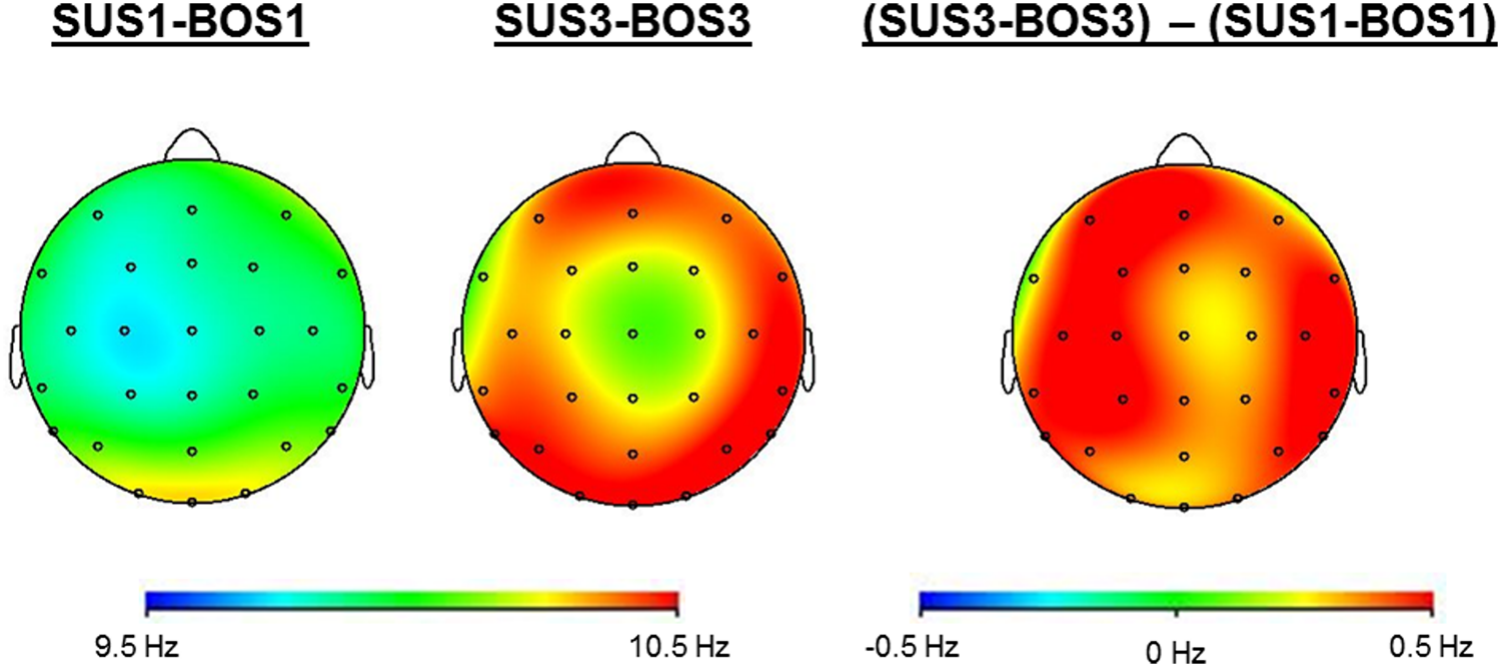
**Cortical mappings indicate iAPF during SUS1-BOS1 and SUS3-BOS3 as well as the difference in iAPF distribution between these two conditions by subtracting iAPF values of SUS1-BOS1 from SUS3-BOS3**.

**Figure 4** shows cortical mappings for iAPF during SUS1-BOS1 and SUS3-BOS3. The three-way ANOVA results for the iAPF are presented in **Table 2**. Significant main effects were found for the factors BOS (*F*_2,72_ = 3.89; *p* = 0.050; η^2^ = 0.10) and ROI (*F*_2,72_ = 8.29; *p* < 0.001; η^2^ = 0.19). *Post hoc* tests indicated an increase in iAPF from bipedal (BOS1) to unipedal stance (BOS2, BOS3). The highest iAPF was observed in the centro-parietal cortex.

**Table 2:** *F*- and *p*-values for the three-way ANOVAs calculated for each frequency band and the iAPF.

	Theta		Lower alpha		Upper alpha		iAPF	
				
	*F*-value	*P*-value	*F*-value	*P*-value	*F*-value	*P*-value	*F*-value	*P*-value
SUS (df = 2,72)	3.12	0.061	**5.10**	**0.008**	1.12	0.333	1.43	0.246
BOS (df = 2,72)	**50.99**	**<0.001**	**11.77**	**<0.001**	0.18	0.834	**3.89**	**0.050**
ROI (df = 2,72)	**4.30**	**0.017**	**9.19**	**<0.001**	**46.95**	**<0.001**	**8.29**	**<0.001**
SUS × BOS (df = 4,144)	**3.07**	**0.018**	1.43	0.227	**2.91**	**0.024**	2.17	0.075
SUS × ROI (df = 4,144)	**3.17**	**0.015**	**3.37**	**0.011**	**3.43**	**0.010**	1.07	0.376
BOS × ROI (df = 4,144)	**6.74**	**<0.001**	**15.18**	**<0.001**	**11.67**	**<0.001**	1.48	0.212
SUS × BOS × ROI (df = 8,288)	0.49	0.80	0.40	0.970	0.30	0.964	0.65	0.739


*Significant main effects and interactions are highlighted in bold type.*

### Spectral Power

**FIGURE 5:**
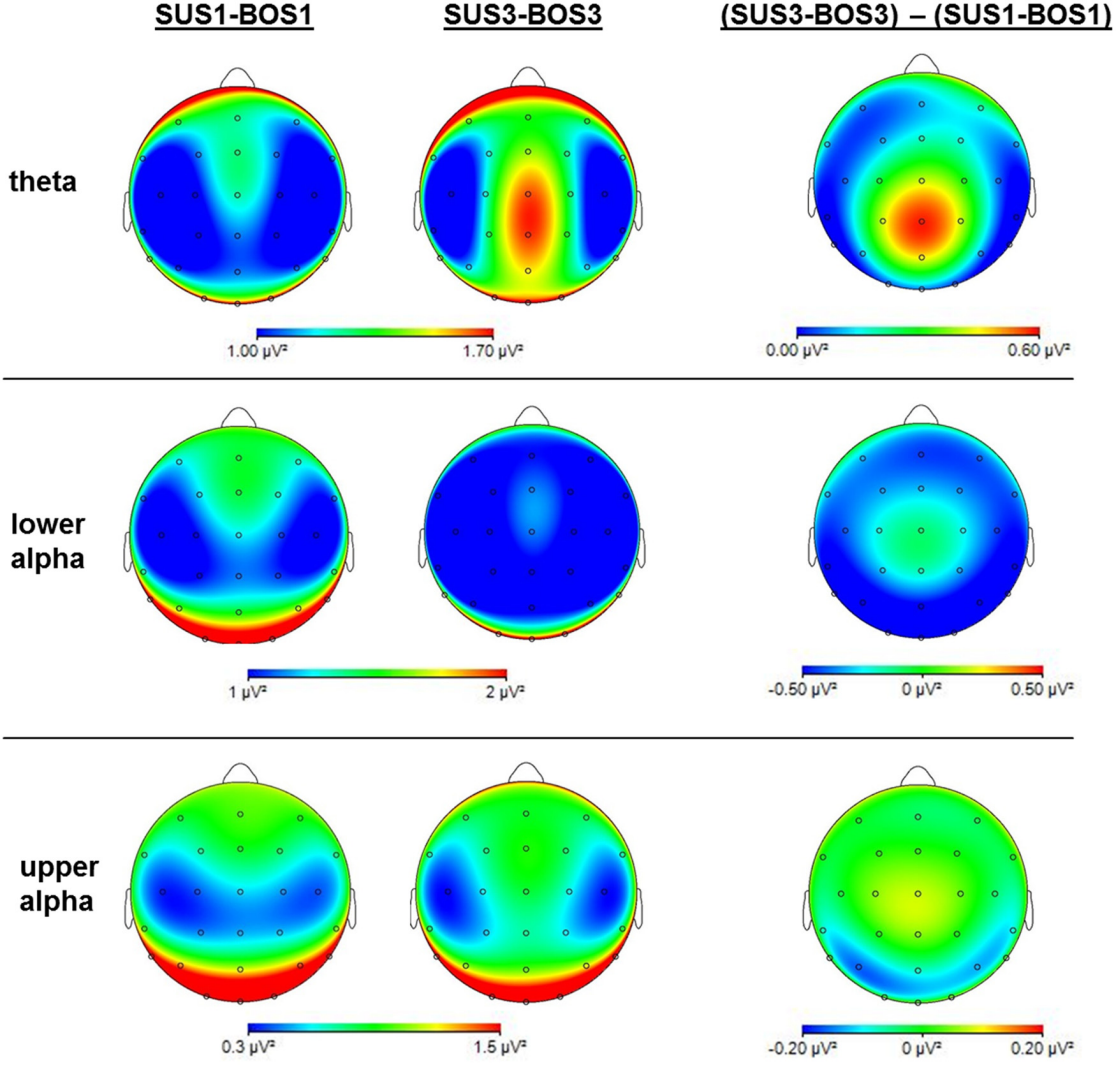
**Mappings of cortical activity for the theta, lower alpha and upper alpha frequency bands.** Mappings indicate cortical activity during SUS1-BOS1 and SUS3-BOS3 as well as the difference in cortical power distribution by subtracting power values of SUS1-BOS1 from SUS3-BOS3. Please note differences in color bar scaling.

The three-way ANOVA results for theta, lower alpha, and upper alpha frequency bands are presented in **Table 2**. In addition, **Figure 5** shows cortical mappings for theta, lower alpha, and upper alpha power during SUS1-BOS1 and SUS3-BOS3 as well as the difference between both conditions (SUS3-BOS3 – SUS1-BOS1).

### Theta Power

In the theta frequency band, the ANOVA yielded significant main effects for BOS (*F*_2,72_ = 50.99; η^2^ = 0.59) and ROI (*F*_2,72_ = 4.30; η^2^ = 0.11). Subsequent *post hoc* analyses revealed an increase in theta activity during unipedal (BOS2, BOS3) stance when compared to bipedal stance (BOS1). The highest theta power was observed in the centro-parietal region. In addition, *post hoc* analysis on the significant interaction for SUS × BOS (*F*_4,144_ = 3.07; η^2^ = 0.08) revealed a stronger increase in theta power from bipedal (BOS1) to unipedal stance (BOS2, BOS3) during SUS2 and SUS3 when compared to SUS1 while the BOS × ROI interaction (*F*_4,144_ = 6.74; η^2^ = 0.16) indicated this increase in theta power to be stronger in the centro-parietal cortex when compared to frontal and fronto-central regions. *Post hoc* analysis investigating the significant SUS × ROI interaction did not reach significance.

### Lower Alpha Power

In the lower alpha frequency band, the three-way ANOVA yielded significant main effects for SUS (*F*_2,72_ = 5.10; η^2^ = 0.12), BOS (*F*_2,72_ = 11.77; η^2^ = 0.25) and ROI (*F*_2,72_ = 9.19; η^2^ = 0.20). *Post hoc* tests indicated a power decrease from SUS1 to SUS3 (*p* = 0.007), as well as from BOS1 to BOS2 (*p* < 0.001) and BOS3 (*p* < 0.001). Further, the lowest lower alpha power was found in the fronto-central region. For both significant interactions, SUS × ROI (*F*_4,144_ = 3.37; η^2^ = 0.09) and BOS × ROI (*F*_4,144_ = 15.18; η^2^ = 0.30), the *post hoc* tests indicated the decrease in lower alpha power induced by altered SUS and BOS was more pronounced in the centro-parietal region.

### Upper Alpha Power

In the upper alpha frequency band, the three-way ANOVA yielded a significant main effect for ROI (*F*_2,72_ = 46.95; η^2^ = 0.57). *Post hoc* tests indicated upper alpha power was highest in the centro-parietal region. In addition, the ANOVA yielded a significant BOS × ROI (*F*_4,144_ = 11.67; η^2^ = 0.24) interaction. *Post hoc* analyses revealed a significant decrease in upper alpha power from BOS1 to BOS3 (*p* < 0.001) in the centro-parietal region. In contrast, upper alpha activity increased from BOS1 to BOS2 in the fronto-central region (*p* = 0.006), however, this increase failed to reach the significance level during BOS3 (*p* = 0.130). There were no changes in the frontal region. *Post hoc* comparisons for the SUS × BOS and SUS × ROI interactions did not reach significance.

### Correlation Analyses

**FIGURE 6:**
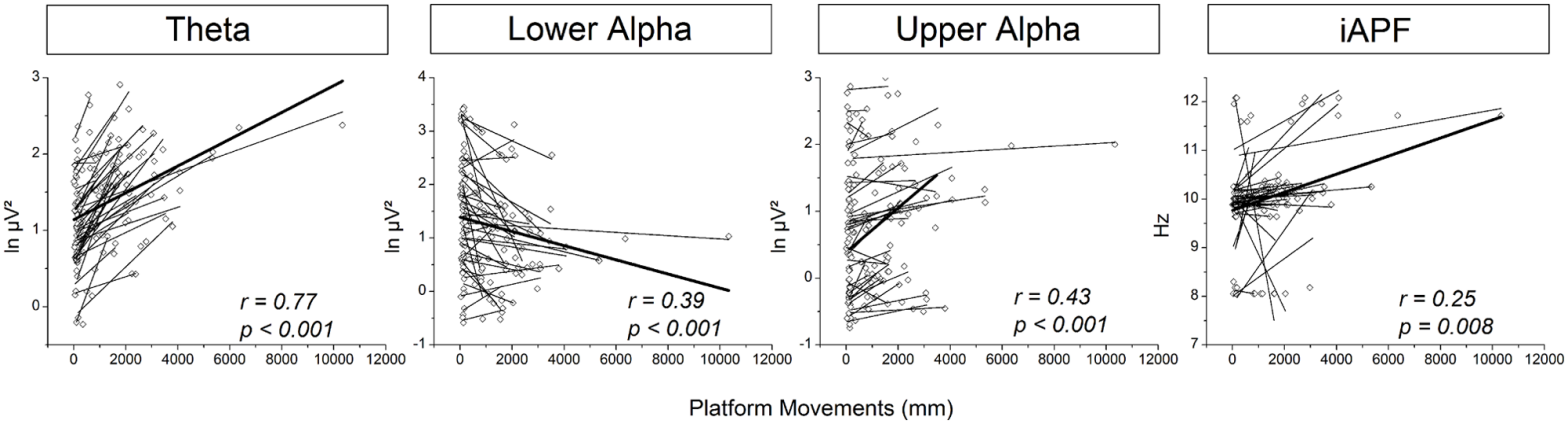
**Correlations between platform movements (*x*-axis) and cortical activity (*y*-axis) displayed from left to right: For theta power on electrode position CPz, lower alpha power on electrode position Pz, upper alpha power on electrode position Cz and iAPF on electrode position CPz.** Dots reflect cortical activity and platform movements for each subject and balance task. Linear fits have been calculated for each subject individually and are displayed as solid lines. The thick line represents the overall linear fit across all subjects and balance tasks. Please note negative spectral power values are due to application of the natural logarithm.

The above described results indicate a ceiling effect for cortical activity as a function of platform movements (i.e., balance task demands). Specifically, despite a very pronounced increase in platform movements during SUS3-BOS2 and SUS3-BOS3 no substantial change in cortical activity was found. Similar results were obtained in the eyes-opened experiment (cf. Hülsdünker et al., 2015). Therefore, these two conditions were excluded from correlational analyses. Correlation coefficients (*r*) were highest for theta power on electrode position CPz (*r* = 0.77; *p* < 0.001). Lower alpha and upper alpha power was best correlated to platform movements on electrode position Pz (*r* = –0.39, *p* < 0.001) and Cz (*r* = 0.43; *p* < 0.001), respectively. Further, a significant correlation between iAPF and platform movements was found for electrode position CPz (*r* = 0.25, *p* = 0.008). These results are illustrated in **Figure 6**. Please note the high intersubject variability with regard to theta power. Therefore, we refrained from correlational analyses between subjects.

## Discussion

The present study is the first to identify changes in cortical activity associated with eyes-closed continuous balance control in a range of tasks varying in difficulty. In accordance with our hypotheses, increasing demands on balance control were accompanied by distinct modulations of theta, lower alpha, and upper alpha cortical activity. In addition, an increase of the iAPF, a neurophysiological marker of thalamo-cortical information transmission, was found during performance of more demanding balance tasks. Detailed Correlation analyses revealed significant relations between platform movements and cortical activity.

### Platform Movements

Balance performance was assessed by means of platform movements of the Posturomed. As expected platform movements were significantly higher when the latch was open and thus, both oscillators were released (SUS3) compared to when it was closed (SUS2). The same holds true for changes in BOS. Platform movements were significantly higher if subjects stood on one leg (BOS2/BOS3) when compared to double-leg stance (BOS1). The largest platform movements were observed during SUS3 while standing on one leg. These results clearly indicate that the chosen balance apparatus is well-suited for measuring individuals’ ability to stand still, and to manipulate balance tasks demands with regard to SUS.

### Individual Alpha Peak Frequency

One important finding of this study is that the iAPF is responsive to increased balance control demands. Specifically, the iAPF increased in frontal, fronto-central and centro-parietal regions of the cortex with higher task demands. The iAPF is a frequency specific component involved in thalamo-cortical information transmission, and therefore, it can be considered as an index of the amount of information that is relayed to the cortex.

From a functional perspective, increased (resting state) iAPF values are directly related to performance in a variety of cognitive tasks (Klimesch, 1999; Richard Clark et al., 2004; Jin et al., 2006). The correlation analysis in this study confirms this relation by indicating lower balance task demands, as reflected by superior balance performance (i.e., less platform movements) to be associated with *lower* iAPF and vice versa. At a first glance, this may appear somewhat surprising, as previous experiments using cognitive tasks reported superior task performance to be accompanied by *higher* iAPF (Richard Clark et al., 2004; Jin et al., 2006). However, it should be noted that previous studies using cognitive tasks correlated iAPF during resting state to cognitive performance whereas in the present study the change in iAPF during actual performance of balance tasks is addressed. Therefore, in this study the iAPF is suggested to reflect a state marker primarily dependent on the individuals’ rate of thalamo-cortical information transmission during task performance that increases with higher demands on balance control. In contrast, resting state iAPF manifests as a stable trait marker (Grandy et al., 2013b) that is strongly dependent on neuroanatomical properties such as white matter integrity (Valdés-Hernández et al., 2010). These differences have to be considered when drawing conclusions on cause and effect of iAPF and balance control. While resting state iAPF may determine task performance (Klimesch, 1999; Richard Clark et al., 2004; Jin et al., 2006), this may be reversed when considering state dependent iAPF where actual task demands in turn determine the iAPF. The latter assumption has previously been confirmed during cognitive tasks where iAPF increased with higher cognitive demands (Haegens et al., 2014; Maurer et al., 2014). Therefore, with regard to the correlation found in the present study it is suggested that lower task demands are accompanied by lower iAPF values as in these tasks the subjects are less demanded and consequently require less information to be relayed to the cortex in order to meet the task demands. Conversely, more challenging balance tasks accompanied by increased platform movements require stronger task engagement to maintain upright posture that increases the amount of thalamo-cortical information transmission facilitated by a higher iAPF.

The above mentioned findings raise the question how an increased iAPF facilitates balance control. Several studies suggest the alpha rhythm to be involved in cortical information processing (Klimesch et al., 2007; Jensen and Mazaheri, 2010; Klimesch, 2012; Grandy et al., 2013a). As information transmission is dependent on alpha phase (Lorincz et al., 2009), an increase in iAPF is suggested to facilitate information processing through more excitation-inhibition cycles per time as well as a better temporal resolution for event detection (Schroeder and Lakatos, 2009; Grandy et al., 2013a). This concept has previously been discussed with regard to cognitive tasks (Haegens et al., 2014) and appears also plausible for the sensorimotor domain. In fact, in a recent study we demonstrated the iAPF is correlated to locomotor skills rather than visual working memory or cognitive processing speed (Mierau et al., 2015a). The results of this study support these findings and suggest an increase in thalamo-cortical information transmission when balance tasks became more demanding.

In sum, the iAPF results of this study provide valuable information and extend previous research focusing on spectral power modulations as the iAPF is a frequency specific component involved in thalamo-cortical information transmission. Importantly, balance task-related modulations in iAPF and spectral power reflect different processing stages of cortical mechanisms involved in balance control. Specifically, modulations in spectral power are suggested to reflect adjustments in task-related cortical resource allocation while the iAPF reflects an upstream process indicating the amount of information that is relayed to the cortex via the thalamus. Therefore, the results of this study indicate higher balance task demands to be not only accompanied by an increase in cortical resource investment and activation (see below) but also by an increase in the overall amount of information relayed by thalamo-cortical connections.

### Theta Power

In the present study, cortical theta activity increased with higher demands on balance control and add further support to the increasing amount of studies emphasizing the involvement of theta oscillations in balance control (Slobounov et al., 2009, 2013; Sipp et al., 2013; Hülsdünker et al., 2015). Specifically, increased theta oscillatory activity in the fronto-central and ACC was previously suggested to subserve the detection and processing of discrepancies between expected and actual state of balance (Slobounov et al., 2009; Sipp et al., 2013) referred to as error-detection and processing (Adkin et al., 2006). We propose similar error detection and processing mechanisms in this study as the magnitude/amount of errors to be detected and processed is suggested to increase with more platform movements. However, the increase in theta power was not restricted to the fronto-central region but was even most pronounced in the centro-parietal cortex. Further support to a centro-parietal contribution to balance control comes from the correlation analyses revealing a strong relation between midline centro-parietal theta activity (electrode position CPz) and platform movements (*r* = 0.77). These results confirm our previous findings on eyes-opened balance tasks (Hülsdünker et al., 2015), however, during eyes-closed balance tasks this relation was considerably stronger when compared to eyes opened conditions (eyes-opened: *r* = 0.37; eyes-closed: *r* = 0.77). This suggests an important role of theta oscillations in somatosensory and/or vestibular information integration to maintain balance in the absence of visual information. In fact, electrode position CPz corresponds best to Brodman Area 5 (BA5) as a part of the posterior parietal cortex (PPC; Koessler et al., 2009) that is established to receive multimodal input from somatosensory, visual and vestibular systems (Andersen et al., 1997) essential to maintain upright posture (Mohapatra et al., 2012; Forbes et al., 2014; Joseph Jilk et al., 2014). During control of balance, the centro-parietal region may be involved in the integration of sensory afferents from the periphery subserving error detection and processing (Sipp et al., 2013; Hülsdünker et al., 2015). In sum, these results suggest a more distributed cortical system involved in error detection and processing that is not restricted to the fronto-central cortex but also contains centro-parietal regions.

### Lower Alpha Power

In addition to changes in the theta frequency band, increasing demands on balance control were accompanied by a widespread decrease of lower alpha activity observed over frontal, fronto-central and centro-parietal areas. Previously, lower alpha power was interpreted to reflect higher levels of information processing (Del Percio et al., 2009). We suggest similar processes in this experiment as afferent sensory information is suggested to increase with more platform movements during more challenging balance tasks. The negative correlation between lower alpha power and balance performance further supports the suggestion of a functional role of lower alpha activity during balance control.

### Upper Alpha Power

In contrast to a widespread decrease in lower alpha, reductions in upper alpha activity were found to be more focally located over the centro-parietal cortex. The increase in fronto-central upper alpha power as well as the positive correlation between upper alpha power on electrode position Cz and platform movements further indicates a functional difference between parietal and central upper alpha activity. As a reduction of upper alpha activity over sensory regions was previously reported during movement execution (Pfurtscheller et al., 2000), the decrease in upper alpha power found in the present study may be attributable to sensory information processing induced by compensatory movements during balance control. The fronto-central increase in upper alpha power as well as the positive correlation in turn may attributable to inhibitory processes (Hummel et al., 2002; Sauseng et al., 2013) during balance control in order to reduce the amount of the degrees of freedom that need to be controlled by the central nervous system. In sum, although there is more research needed, the combined pattern of result on alpha activity demonstrates the involvement of alpha oscillations in balance control processes. Further, functional differences between lower and upper alpha rhythms highlight the importance of differentiation between these two frequency bands.

### Methodological Remarks and Limitations

The results and interpretations from this study should be evaluated within the context of its methodological framework. Specifically, the here reported modulations in lower and upper alpha power could potentially be related to the shift in iAPF. However, the change in iAPF is unlikely to explain spectral power modulations in the alpha band as the decrease in the lower alpha power was global whereas changes (i.e., increase and decrease) in the upper alpha frequency band were regionally distinct. Nevertheless, potential interactions between iAPF and spectral power should be addressed in future studies.

Another methodological aspect that deserves critical discussion is whether a common average used on a 32 channel recording is appropriate when associating electrode activity with activity from cortical areas. This is because prominent activity in one region may be mirrored into relatively calm regions due to the high impact of high amplitude oscillations when using a relatively low number of channels. However, it must be noted that the present study focuses on topographical *differences* between conditions rather than on topographical distribution of activity within a single condition. This approach should substantially reduce the risk for a systematic error due to the average reference procedure.

Finally, it should be acknowledged that although EEG is generally limited by its spatial resolution more recent source localization algorithms, such as LORETA (Pascual-Marqui et al., 2011) or dipole fitting (Oostenveld and Oostendorp, 2002), can provide reasonable source localization results (Slobounov et al., 2009; Gwin et al., 2011; Sipp et al., 2013). The precision of the localization of the EEG signal using these techniques increases with increasing number of channels (Sohrabpour et al., 2015), and further localization improvements can be achieved by projecting the EEG channels onto a magnetic resonance image (MRI) of the individuals’ brain (Michel and Murray, 2012). Therefore, future studies addressing the neuroanatomical correlates of postural control and balance would benefit from a larger number of channels and additional MRI scans of the individual’s brain.

## Conclusion

The results of the present study indicate a global increase in iAPF with higher demands on balance control. We suggest a higher iAPF to reflect an increase in information transmission during higher task demands. Furthermore, higher midline theta activity was found to be strongly correlated to platform movements probably reflecting a stronger engagement in error detection and processing due to increased balance task demands. Differential behavior of lower and upper alpha sub-bands is suggested to reflect a global increase in cortical activation and motor inhibition necessary to reduce the degrees of freedom, respectively. The combined pattern of results in this study provides new insights into spectral and spatial characteristics of cortical oscillations subserving balance control. Specifically, while modulations in spectral power are suggested to reflect adjustments in cortical resources invested during balance control, the increase in iAPF further indicate an increase in overall thalamo-cortical information transmission when balance tasks become more challenging. This information may be particularly useful in a clinical context as they could be used to reveal cortical contributions to balance dysfunction in specific populations such as Parkinson’s or vestibular loss. However, this is a hypothesis to be tested in future studies rather than a final conclusion.

## Author Contributions

TH, AM, and HS were responsible for design and concept of the study. TH and AM acquired and analyzed data, and were responsible for interpretation of findings. TH and AM drafted the manuscript. All authors critically reviewed the manuscript for important intellectual content and approved the final version for publication.

## Conflict of Interest Statement

The authors declare that the research was conducted in the absence of any commercial or financial relationships that could be construed as a potential conflict of interest.
