# Supplementary material for: Higher Balance Task Demands are Associated with an Increase in Individual Alpha Peak Frequency
Source: Front Hum Neurosci. 2016 Jan 6;9:695. doi: 10.3389/fnhum.2015.00695 (PMC4702132; doi:10.3389/fnhum.2015.00695)
Supplement: Supplementary file 1 [file Table_1.PDF]

Supplementary table 1: Means (±95% confidence intervals) of oscillatory components (ln-transformed frequency band power, iAPF) and platform movements for all balance tasks. Please note balance tasks during SUS1 were performed on solid surface hence there are no platform movement data for the SUS1 condition.

Oscillatory component	Region of interest (ROI)	SUS1			SUS2			SUS3		
		BOS1	BOS2	BOS3	BOS1	BOS2	BOS3	BOS1	BOS2	BOS3
theta (ln µV²)	frontal	1.15(±0.13)	1.34(±0.18)	1.36(±0.20)	1.00(±0.15)	1.27(±0.15)	1.30(±0.15)	1.04(±0.16)	1.34(±0.17)	1.38(±0.15)
	fronto-central	1.04(±0.14)	1.20(±0.16)	1.19(±0.17)	0.91(±0.15)	1.22(±0.14)	1.22(±0.13)	0.96(±0.15)	1.26(±0.14)	1.26(±0.14)
	centro-parietal	1.04(±0.17)	1.34(±0.17)	1.30(±0.20)	0.92(±0.16)	1.33(±0.15)	1.32(±0.14)	0.96(±0.15)	1.40(±0.16)	1.43(±0.16)
lower alpha (ln µV²)	frontal	1.33(±0.31)	1.09(±0.32)	1.02(±0.31)	1.21(±0.32)	0.98(±0.29)	1.01(±0.30)	1.11(±0.31)	1.02(±0.30)	0.95(±0.25)
	fronto-central	1.16(±0.29)	0.94(±0.31)	0.90(±0.30)	1.09(±0.30)	0.93(±0.30)	0.97(±0.30)	1.02(±0.29)	0.90(±0.28)	0.87(±0.26)
	centro-parietal	1.43(±0.33)	0.10(±0.30)	0.97(±0.30)	1.39(±0.33)	0.98(±0.29)	1.03(±0.29)	1.27(±0.32)	0.93(±0.28)	0.93(±0.25)
upper alpha (ln µV²)	frontal	0.91(±0.35)	1.00(±0.32)	0.93(±0.34)	0.93(±0.35)	0.84(±0.33)	0.92(±0.33)	0.93(±0.35)	0.87(±0.30)	0.84(±0.31)
	fronto-central	0.62(±0.32)	0.79(±0.32)	0.75(±0.33)	0.70(±0.32)	0.70(±0.31)	0.81(±0.32)	0.69(±0.33)	0.60(±0.30)	0.70(±0.32)
	centro-parietal	1.08(±0.35)	1.03(±0.33)	1.03(±0.35)	1.18(±0.36)	1.01(±0.36)	1.10(±0.36)	1.15(±0.35)	0.96(±0.32)	0.96(±0.31)
iAPF (Hz)	frontal	9.91(±0.21)	10.18(±0.32)	10.39(±0.34)	10.20(±0.29)	10.14(±0.33)	10.26(±0.31)	10.07(±0.29)	10.26(±0.32)	10.40(±0.30)
	fronto-central	9.82(±0.20)	10.06(±0.33)	10.27(±0.31)	9.97(±0.31)	10.18(±0.31)	10.21(±0.29)	9.89(±0.26)	10.09(±0.34)	10.25(±0.26)
	centro-parietal	9.98(±0.14)	10.27(±0.31)	10.35(±0.29)	10.19(±0.22)	10.37(±0.28)	10.26(±0.29)	10.20(±0.20)	10.39(±0.30)	10.41(±0.27)
platform movements (mm)					102.02 (±16.18)	2046.62 (±449.84)	2201.77 (±580.33)	333.80 (±183.36)	7957.84 (±943.33)	8022.51 (±891.14)
